# Ampullary Neuroendocrine Tumors: Multicenter Experience and Emerging Perspectives on Endoscopic Treatment

**DOI:** 10.5041/RMMJ.10562

**Published:** 2026-01-28

**Authors:** Federica Fimiano, Lorenzo Dioscoridi, Marta Stegagnini, Edoardo Forti, Francesco Pugliese, Marcello Cintolo, Giulia Bonato, Marianna Bravo, Andrea Palermo, Camilla Gallo, Cecilia Binda, Claudio Zulli, Piera Zaccari, Alberto Mariani, Armando Gabbrielli, Paolo Giorgio Arcidiacono, Massimiliano Mutignani

**Affiliations:** 1 Digestive Endoscopy Unit, AOU San Giovanni e Ruggi-Fucito Unit, Salerno, Italy; 2 Digestive Endoscopy Unit, ASST Niguarda, Milan, Italy; 3 Gastroenterology and Digestive Endoscopy Unit, Forlì-Cesena Hospitals, Romagna, Italy; 4 Pancreato-Biliary Endoscopy and Endosonography Division, Pancreas Translational & Clinical Research Center, San Raffaele Scientific Institute IRCCS, Vita-Salute San Raffaele University, Milan, Italy; 5 Diagnostic and Interventional Endoscopy of Pancreas, Pancreas Institute, University of Verona, Verona, Italy

**Keywords:** Ampullary NETS, endoscopy, ERCP, NETs, papillectomy

## Abstract

**Background:**

Ampullary neuroendocrine tumors (NETs) are extremely rare, representing 0.3%–1% of gastrointestinal NETs and less than 2% of periampullary cancers. Due to their rarity, there is limited data on their natural history, management, and outcomes. Current European Neuroendocrine Tumor Society guidelines (2023) recommend pancreaticoduodenectomy (PD) as the standard treatment. However, this approach is invasive and associated with high morbidity and mortality. Emerging evidence suggests that endoscopic papillectomy (EP) could be a viable alternative in selected cases. This retrospective multicenter study aimed to evaluate the feasibility and outcomes of endoscopic resection for ampullary NETs.

**Methods:**

This retrospective case series included 14 patients who underwent EP for ampullary NETs between 2011 and 2022 across three Italian tertiary centers. Pre-procedural evaluation was performed following European Society of Gastrointestinal Endoscopy guidelines. Endoscopic papillectomy was performed under monitored sedation, using standard snares for *en bloc* resection. Follow-up endoscopy was conducted at a median of 3 months. Primary outcomes included complete resection (R0) and recurrence rates; secondary outcomes focused on adverse events.

**Results:**

Fourteen patients (median age: 62.5 years; 50% male) were included. Median tumor size was 18 mm. In 12 out of 14 cases, ampullary NETs were diagnosed only after endoscopic resection. Post-resection histology identified 8 G1 NETs (Ki-67 1%) and 6 G2 NETs (Ki-67 5%). Complete resection was achieved in 11 cases (78.6%). Among 3 incomplete resections, 2 were managed surgically, while 1 was followed up without recurrence. Residual disease was detected in 3 patients: 2 were managed endoscopically, and 1 required surgery. No recurrences occurred during a median follow-up of 14.5 months. Adverse events occurred in 42.9% of patients, including 5 cases of bleeding and 1 case of mild pancreatitis, all resolved without major sequelae. Median hospital stay was 2.5 days.

**Conclusions:**

Our findings suggest that EP offers a promising alternative to surgery in selected patients with ampullary NETs. Endoscopic resection was associated with high rates of R0 and favorable short-term outcomes, with effective endoscopic management of residual disease and procedure-related adverse events. Consistent post-procedural surveillance remains essential to detect residual or recurrent disease. Larger prospective studies are warranted to refine patient selection criteria, optimize protocols, and establish the long-term efficacy.

## BACKGROUND

Ampullary neuroendocrine tumors (NETs) are exceptionally rare, accounting for only about 0.3%–1% of all gastrointestinal neuroendocrine tumors and less than 2% of all periampullary cancers.^1^ Due to their rarity, there is limited data on their natural history, optimal management, and long-term outcomes. According to the 2023 European Neuroendocrine Tumor Society (ENETS) guidelines, the recommended standard treatment for periampullary NETs is surgical resection via pancreaticoduodenectomy (PD).^2^ This approach is considered the procedure of choice for ampullary and periampullary neoplasms due to their aggressive nature and the lack of consistent data on their behavior in the literature.^3^ Furthermore, ampullary NETs have a higher proportion of grade 3 (G3) tumors compared to duodenal NETs, exhibit more frequent lymph node metastases, and demonstrate a lower 5-year overall survival rate compared to patients with duodenal NETs. However, while PD is effective, it is also an invasive procedure associated with significant morbidity and mortality.^4^ Emerging data on endoscopic treatment offer promising alternatives, though further research is needed to validate these less invasive strategies.

This study aimed to summarize our experience with endoscopic resection for ampullary NETs by assessing the rate of complete resection (R0) and analyzing recurrence rates. Secondary outcomes included post-procedural adverse events (AEs), thus providing a comprehensive understanding of the safety and feasibility of this therapeutic approach.

## MAERIALS AND METHODS

We conducted a multicenter retrospective cohort study analyzing patients who underwent endoscopic papillectomy (EP) for ampullary NETs from 2011 to 2022 across three tertiary centers in Italy. During this period, 14 patients underwent endoscopic treatment and were included in the study. Data were collected from endoscopy databases and electronic medical records, including patient demographics (sex, age), symptoms at diagnosis, histology before and after EP (available for all patients), AEs, recurrence rates, retreatment strategies, need for surgery, and survival outcomes. All lesions were confined to the papilla, as confirmed by pre-procedural imaging. Informed consent was obtained from all patients regarding procedure modalities and associated risks. The study was conducted in accordance with regional ethical governance and with the approval of the primary coordinating institutional review board (protocol number: 2023-EP-73).

Comprehensive pre-procedural evaluation was completed for all patients, including endoscopic ultrasound (EUS) or magnetic resonance imaging (MRI), to assess local tumor stage, regional lymph node involvement, and the extent of intraductal infiltration, in accordance with European Society of Gastrointestinal Endoscopy (ESGE) guidelines.^5^ All procedures were performed under monitored deep sedation by experienced high-volume endoscopists with at least 10 years of experience at the start of the study (2010).

Duodenoscopes with a 4.2-mm therapeutic working channel were used, and endoscopic retrograde cholangiopancreatography was attempted in all cases. Pancreatitis prophylaxis was carried out using rectal diclofenac or indomethacin. Endoscopic papillectomy was the primary treatment modality and was performed using *en bloc* resection with standard polypectomy snares. The procedure utilized “Endocut Q” electrosurgical mode (effect 3, cut duration 1; ERBE, Tübingen, Germany) to ensure precise and controlled tissue resection.

In most cases, the placement of pancreatic (5–7 Fr, 5 cm) and biliary stents (8.5–10 Fr, 5 cm with flaps) was intended to prevent secondary fibrotic strictures, although it was not always feasible. Endoclips were used to close mucosal defects whenever possible to reduce the risk of post-procedural bleeding. Periprocedural management of antiplatelet and anticoagulant therapies followed standard guidelines for high-risk endoscopic procedures.^6^

Resection was classified as R0 when histopathological analysis confirmed negative margins on the resected specimen. It was classified as incomplete resection (R1) if microscopic residual tumor was present at the resection, and further incomplete resection (R2) in cases where macroscopic residual tumor was left behind, either visible during the procedure or identified on pathological evaluation.^5^,^7^

The 2023 ENETS guidelines for NETs grading classify tumors as follows: Grade 1 (G1), Ki-67 index <3%; Grade 2 (G2), Ki-67 index between 3% and 20%; and Grade 3 (G3), Ki-67 index >20%.^2^

Adverse events were categorized based on ESGE guidelines,^8^ including acute pancreatitis, post-procedural bleeding, perforation, and acute cholangitis within 30 days post-procedure. Acute pancreatitis was defined according to the revised Atlanta criteria.^9^ Duodenal perforation was diagnosed by direct endoscopic/fluoroscopic visualization or by the presence of contrast leakage and/or free fluid/abscesses on follow-up CT scans. Post-procedural bleeding was defined as melena requiring blood transfusion or endoscopic, surgical, or angiographic intervention. Acute cholangitis and cholecystitis were defined per the Tokyo Guidelines 2018.^10^

After EP, patient monitoring included duodenoscopy with biopsies of the scar and any abnormal area at 3 months, followed at 6 and 12 months, and annually thereafter for at least 5 years if no recurrence was detected. Additional imaging with MRI or EUS was performed when clinically indicated, based on initial tumor characteristics or findings during follow-up. In case recurrence occurred and was endoscopically treated, follow-up was intensified with endoscopic reassessment every 3 months. Residuals and recurrences were treated by bipolar intraductal radiofrequency ablation (7 W, 90 s) or snare resection (hot or cold) or with pancreaticoduodenectomy when deemed necessary. Prior to radiofrequency ablation, pancreatic stenting (5–7 Fr, 5 cm) was routinely performed, while biliary stenting (10 Fr 5 cm plastic or 10 mm 4 cm fully covered self-expanding metal stent) was placed at the end of the procedure.

### Statistical Analysis

Descriptive statistics were calculated as either mean values with standard deviation (SD) or median values with interquartile range (IQR), depending on the distribution of the data.

## RESULTS

Fourteen patients met the eligibility criteria during the study period. Patient demographics and ampullary NET characteristics are summarized in Table 1. Seven patients were symptomatic at diagnosis. Preoperative staging was performed using MRI (4 patients), EUS and contrast enhanced-EUS (CH-EUS) (8 patients), or both (2 patients), and in all cases both modalities suggested the presence of an ampullary tumor. A dilated main pancreatic duct (MPD) (>5 mm) was observed in 2 patients (14.3%), while a dilated common bile duct (CBD) was identified in 5 patients (35.7%). Intraductal extension, with a maximum length of 14 mm, was documented in 3 patients (21.4%), involving the CBD in 2 cases and the MPD in 1 case.

**Table 1:** Baseline Characteristics of Study Population and Ampullary NETs.

Parameter	Overall (*n*=14)	% or IQR
Population characteristics		
Age (year, median)	62.5	52.3–72.8

Sex (M/F)	7/7	50%/50%

Pre-endoscopic staging		
Lesion size (mm, median)	18	15–24.5

Main pancreatic duct dilation (>5 mm)	2	14.3%

Bile duct dilation	5	35.7%

Intraductal extension	3	21.4%

Intraductal extension (mm, median)	5	3–14

Histology on pre-papillectomy biopsies		
LGD adenoma	4	28.6%

HGD adenoma	1	7.14%

Adenocarcinoma	0	0%

NET (non-functional)	2	14.3%

Other (non-specific findings)	7	50%

HGD, high-grade dysplasia; LGD, low-grade dysplasia; NET(s), neuroendocrine tumor(s).

Preoperative biopsies revealed low-grade dysplasia in 4 cases, high-grade dysplasia in 1 case, NETs in only 2 cases, and non-specific findings in 7 cases.

The diagnosis of ampullary NET was established only after EP in 12 out of 14 patients. In the remaining 2 cases, EP was performed despite a preoperative histological diagnosis of NET, as 1 patient was deemed unfit for surgery and underwent an endoscopic approach, while the other opted for endoscopic intervention over surgery due to personal preference. These 2 patients underwent a Gallium-68 positron emission tomography (PET) scan, which showed tracer uptake only at the papillary level. Histological analysis of the resected specimens confirmed the presence of NETs in all patients (100%), with NET G1 (Ki-67 <3%) in 8 cases and NET G2 (Ki-67 3–20%) in 6 cases (Table 2). The median size of the ampullary NETs was 18 mm (IQR 15–24.5). None of the histological specimens showed lymphovascular invasion.

**Table 2:** Treatment and Histological Outcome Following Endoscopic Resection of Ampullary NETs.

Parameter	Overall (*n*=14)	%
Treatment		
*En bloc* resection	13	92.9%
IDE treatment	1	7.14%

Histology on papillectomy		
NET	14	100%

NET grading		
G1 (Ki-67 <3%)	8	57.1%
G2 (Ki-67 3%–20%)	6	42.9%
G3 (Ki-67 >20%)	0	0%

Resection margin		
R0	11	78.6%
R1	2	14.3%
R2	1	7.14%

G1, grade 1 (Ki-67 <3%); G2, grade 2 (Ki-67 3%–20%); IDE, intraductal extension; NET, neuroendocrine tumors; R0, complete resection; R1, incomplete resection with microscopic residue; R2, incomplete resection with macroscopic residue.

Complete resection was achieved in 11 out of 14 patients (78.6%). Among the 3 patients who did not achieve R0, 2 had R1. Of these, 1 underwent PD, with histological examination of the surgical specimen revealing no residual tumor cells, while the other opted for close follow-up and showed no evidence of recurrence after 24 months of monitoring. The third non-R0 patient had an incomplete R2 and subsequently underwent PD following the initial endoscopic procedure.

After 3 months of follow-up, residual ampullary NETs were identified in 3 out of 11 patients (27.3%) who remained at risk for residual untreated disease. Two of these patients underwent endoscopic management. The first patient, diagnosed with a G1 NET, underwent successful treatment in a single session using hot snare resection to remove the residual le-sion. The second patient, with a G2 NET, was managed with cold snare resection followed by four sessions of bipolar intraductal radiofrequency ablation (7 W, 90 s) to address intraductal residual tissue, based on pathology reports. The third patient, also diagnosed with a G2 NET, underwent PD. The median follow-up duration was 14.5 months (IQR 5–26 months), and no evidence of recurrence was observed at the last follow-up.

The overall AEs rate was 42.9% (6/14 patients). Among the early complications (within 24 hours of the procedure), there were 5 cases of bleeding, 2 mild and 3 moderate, all of which were managed endoscopically. Additionally, there was 1 case of mild acute pancreatitis, treated conservatively. Regarding late complications, one of the patients who had previously experienced moderate bleeding developed recurrent bleeding 24 hours after endoscopic treatment and required radiological embolization. The patient with mild acute pancreatitis subsequently developed severe bleeding 12 days after the procedure, which was managed endoscopically (Table 3). The median hospital stay was 2.5 days (IQR 1–5 days).

**Table 3:** Post-procedural outcomes in EP of ampullary NETs.

Parameter	Overall (*n*=14)	% or IQR
Post-procedural AEs		
Number of patients	6	42.9%
Bleeding	5	83.33%
Acute pancreatitis	1	16.67%

Surveillance		
Residual disease on follow-up	3/11	27.3%

Residual disease management		
Endoscopic resection	2	14.3%
RFA	1	7.14%
PD	1	7.14%

Disease recurrence	0	0%

Median follow-up (months)	14.5	5–26

Total number of patients who underwent PD	3	21.4%

AE, adverse event; EP, endoscopic papillectomy; NETs, neuroendocrine tumors; PD, pancreaticoduodenectomy; RFA, radiofrequency ablation.

## DISCUSSION

Due to their infrequent occurrence and the limited number of reported cases, there is a lack of robust data regarding natural history, optimal management strategies, and long-term outcomes of ampullary NETs. In our multicenter study, we tried to highlight diagnostic challenges and therapeutic approaches for these rare tumors.

Remarkably, in our patient series, only 2 out of 14 cases were diagnosed with ampullary NET prior to papillectomy. This highlights the diagnostic challenges associated with these lesions when performing gastroscopy/duodenoscopy. As NETs originate from the deep mucosa or submucosa, the diagnostic yield of biopsies performed is low, ranging from 14% to 66%.^11^–^14^

The 2023 ENETS guidelines recommend surgical intervention with PD as the gold-standard treatment for periampullary NETs.^2^ However, this highly invasive procedure is associated with significant morbidity, including a notable complication rate and high mortality.^4^,^15^,^16^ Our case series suggests that, in carefully selected patients, endoscopic management can be a feasible and effective alternative. The feasibility, efficacy, and safety of endoscopic resection for ampullary NETs partly builds on the broader experience with duodenal NETs and insights from small cohorts or case series focused on ampullary NETs.^17^–^19^

The feasibility and safety of achieving R0 for ampullary NETs are supported by case series and reports involving both ampullary and non-ampullary duodenal NETs.^17^–^19^ Despite the inherent aggressiveness of ampullary NETs, the higher reported rates of lymph node metastasis, and poorer prognosis compared to duodenal NETs,^3^ our study observed a relatively favorable outcome with endoscopic resection. Complete resection was achieved in 11 of the 14 patients (78.6%). Of the two patients with an R1 margin, the one who underwent PD showed no evidence of residual disease in the surgical specimen, while the patient managed with follow-up experienced no recurrence. This highlights the uncertainty in interpreting R1 findings for this type of NETs, as they may be attributable to artifacts on the histological specimen caused by the resection procedure on the pseudocapsule in the deep planes of the NET. Notably, despite being classified as R1, these patients did not develop local recurrence during follow-up. Therefore, the decision to pursue further radical intervention should be carefully weighed, taking into account the procedure-related risks of complications, patient comorbidities, and age.^20^,^21^ This tailored approach ensures that therapeutic strategies align with the overall clinical picture and patient-specific factors.

Several studies have shown that the prognosis of ampullary NETs is significantly influenced by factors such as tumor size and grading. Specifically, tumors larger than 20 mm and those with a G3 grade are associated with poorer outcomes compared to smaller, lower-grade lesions (G1/G2).^17^,^22^,^23^ In our case series, all the ampullary NETs were localized to the papilla, with a median size of 18 mm. Furthermore, all cases had a low grading (G1 or G2), which is consistent with a better prognosis compared to G3 tumors. These findings suggest that, in our cohort, the tumors exhibited favorable characteristics in terms of both size and grading, which may have contributed to the overall positive outcomes observed.

On 3-month follow-up endoscopy, residual disease was found in only 3 patients (27.3%). Two of these patients were successfully treated with endoscopic methods, while only one required additional surgical intervention. Notably, at a median follow-up of 14.5 months, no recurrences were noted in the patients who received endoscopic treatment, suggesting that in carefully selected cases, endoscopic resection may provide adequate local control without the need for radical surgery.

As for AEs, the bleeding rate was notably higher than typically observed.^24^ This discrepancy can likely be attributed to the peculiar characteristics of NETs, which are highly vascularized and located in the submucosa.^25^,^26^ These features increase the risk of bleeding during resection procedures and may explain the elevated complication rate observed in our cohort.

Our study’s limitations include its retrospective design, the relatively small sample size, and the short follow-up duration for some patients. The limited number of cases, while reflective of the rarity of ampullary NETs, restricts the chance to generalize our findings.

## CONCLUSION

While surgical resection remains the standard of care for ampullary NETs, our findings support the feasibility of endoscopic resection in selected patients. Endoscopic techniques were associated with high rates of R0 and favorable short-term outcomes, with effective endoscopic management of residual disease and complications. Although R0 is generally considered important for optimal local control based on existing evidence, our study was not powered to compare outcomes between R0 and R1 due to the small study population. Consistent and structured post-procedural surveillance remains essential to detect and manage any residual or recurrent lesions. Larger prospective studies are needed to better define the role of endoscopic therapy in the management of ampullary NETs, refine patient selection criteria, and develop clearer evidence-based clinical guidelines.

## Abbreviations

AEs: adverse events
ENETS: European Neuroendocrine Tumor Society
EP: endoscopic papillectomy
ESGE: European Society of Gastrointestinal Endoscopy
EUS: endoscopic ultrasound
IQR: interquartile range
MRI: magnetic resonance imaging
NETs: neuroendocrine tumors
PD: pancreaticoduodenectomy
R0: complete resection
SD: standard deviation
